# Measuring the Pupillary Light Reflex Using Portable Instruments in Applied Settings

**DOI:** 10.3390/vision8040060

**Published:** 2024-10-01

**Authors:** Nicola S. Gray, Menna Price, Jennifer Pink, Chris O’Connor, Ana Antunes, Robert J. Snowden

**Affiliations:** 1 Department of Psychology, Swansea University, Swansea SA2 8PP, UK; nicola.s.gray@swansea.ac.uk (N.S.G.);; 2 Swansea Bay University Health Board, Bridgend CF31 4LN, UK; 3 Aneurin Bevan University Health Board, Newport NP18 3XQ, UK; 4 School of Psychology, Cardiff University, Cardiff CF10 3AT, UK

**Keywords:** age, anxiety, parasympathetic nervous system, pupillometry, trauma

## Abstract

The early components of the pupillary light reflex (PLR) are governed by the parasympathetic nervous system. The use of cheap, portable pupillometry devices may allow for the testing of parasympathetic-system health in field settings. We examined the reliability of two portable instruments for measuring the PLR and their sensitivity to individual differences known to modulate the PLR. Parameters of the PLR were measured in a community sample (*N* = 108) in a variety of field settings. Measurements were taken using a commercial pupillometer (NeuroLight, IDMED) and an iPhone using the Reflex Pro PLR analyser (Brightlamp). The parameters of baseline pupil diameter, constriction latency, amplitude and relative amplitude of constriction, and constriction velocity were measured. Individual differences related to age, levels of anxiety, and post-traumatic stress disorder (PTSD) symptomology were assessed. Some measures could not be attained using the iPhone under these field conditions. The reliability of the measures was high, save for the measurement of contraction latency which was particularly unreliable for the iPhone system. The parameters of the PLR showed the same internal relationships as those established in laboratory-based measurements. Age was negatively correlated with all the reliable PLR parameters for both systems. Effects of anxiety and PTSD symptomology were also apparent. The study demonstrated that a hand-held portable infrared pupillometer can be used successfully to measure the PLR parameters under field settings and can be used to examine individual differences. This may allow these devices to be used in workplaces, sports fields, roadsides, etc., to examine parasympathetic activity where needed.

## 1. Introduction

The eyes have often been thought of as a porthole to the workings of the brain, with with variations in pupil size being associated with an array of processes [1]. Over the past 50 years, interest in this assay of mental life has increased dramatically, and the use of pupillometry, and the pupillary light reflex (PLR) in particular, has been expanded into many clinical and applied settings, including measurement of individual differences [2,3,4,5,6,7,8,9].

### 1.1. The Pupillary Light Reflex

The size of the pupil at any moment in time reflects a balance between the activity of two muscles in the eye, the sphincter and the dilator. These two muscles are controlled via different systems, with the sphincter muscle (whose activation causes the pupil to constrict) being under the control of the parasympathetic branch of the autonomic nervous system (PNS) and the dilator (whose activation causes a dilation of the pupil) under the control of the sympathetic branch of the autonomic nervous system (SNS). The size of the pupil reflects a balance between these two systems.

**Figure 1:**
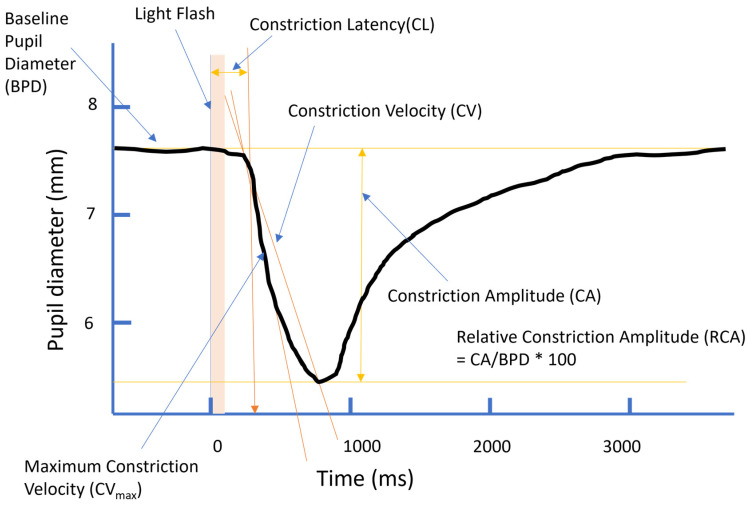
Illustration of the pupillary light reflex and measurement parameters.

Following a sudden increase in light (or a flash of light), the PNS reacts quickly to constrict the pupil, followed somewhat later by the SNS that serves to re-dilate the pupil. This interplay between the two systems produces the prototypical PLR illustrated in Figure 1. Crucially, for the purpose of this study, the early constriction of the pupil in response to a flash of light is thought to be mediated purely by the action of the PNS [10,11]. Hence, measurement of these early components isolates PNS activity.

### 1.2. Parasympathetic Nervous System and Health

While the SNS is known for its reaction to acute stressors (“fight vs. flight”), the PNS serves to recover from stress and restore energy. Dysfunction of the PNS is associated with long-term physical and mental health problems [12,13] including, but not limited to, anxiety disorders/depression [14,15], PTSD [16], and current fatigue/burnout [17]. Further, therapies aimed at calming people and relaxation can also increase PNS activity [18,19]. Hence, the measurement of PNS function can be most valuable in assessing a range of psychological indicators of well-being and could be used to monitor mental health deterioration and therapies.

### 1.3. Measuring the PLR

Traditionally, pupil response was measured qualitatively by experienced clinicians using a simple penlight. The development of infrared cameras has led to more precise measurement of the PLR, and therefore, quantitative assessments of function. However, these systems are expensive and not portable and can require trained operatives. Hence, the majority of studies using pupillometry have been performed in laboratory settings where the participant is dark-adapted and light level is well controlled. However, given the many possible uses of pupillometry, it would be desirable to have instruments that could be used in applied settings, such as the home, workplace, sports field, or roadside.

More recently, less expensive and more available instruments have been used to measure the PLR [9,17,20,21,22,23,24]. In particular, software has been developed that runs on the iPhone in conjunction with the camera to record the pupil response as well as the flash-light to deliver the light pulse. While early reports suggest good agreement between measurements taken using the iPhone system and a laboratory-based infrared camera system [21], other reports [23] have noted high rates of “failure” to take readings and a lack of agreement in the dynamic measures between the iPhone and other portable infrared pupillometry devices.

Given the portable nature of such modern assessment systems, there is the hope that such measurements might be used in more applied settings. For instance, the use of PLR is being explored to look at possible drowsiness or intoxication in drivers [25] and in work situations where high alertness is needed [4], as well as in clinical settings [5,6].

While there are some studies of the relationship between these portable pupillometers and measurements taken with more traditional laboratory-based instruments, there appear to be no studies on the reliability of such measurement, or whether such portable pupillometers can also measure individual differences, in applied/field settings.

### 1.4. Components of the PLR

The PLR can be quantified in various ways (see Figure 1). Just before the flash of light, the pupil will be at a resting level, termed the baseline pupil diameter (BPD). The BPD is thought to be governed by the interplay of both the PNS and the SNS [26] and is therefore not strictly a measure of PNS activity in isolation. Shortly after the light stimulus (typically 200–300 ms), the pupil begins to constrict, and this time is termed the “constriction latency” (CL). The constriction phase then takes approximately 400–600 ms, resulting in a nadir (or minimum diameter) at around 800–1200 ms. This constriction phase can be quantified in various ways. Perhaps the simplest is the “constriction amplitude” (CA) of the response, which is the difference between the BPD and the smallest size reached (minimum diameter). However, as this amplitude is known to be dependent on the initial size of the pupil (the BPD), many researchers recommend using the “relative constriction amplitude” (RCA), which is the percentage change in the diameter of the pupil with respect to the BPD. Other studies have quantified this response in terms of its “speed” rather than its amplitude. This can be done via the measurement of “constriction velocity” (CV), which calculates the speed from the beginning of the response (the response latency) to the point of minimum diameter. Alternatively, some equipment attempts to measure the “maximum constriction velocity” (CV_max_), which is the maximum speed achieved by the pupil during its constriction. Crucially, all these measures of constriction are thought to rely solely on activity in the PNS, with little influence from the SNS.

The activity of the PNS can thus be indexed via any of the measures of the early constriction response outlined above, and analyses have shown a high degree of correlation between them [17]. However, there is currently little information as to which of these parameters is most reliable, the effects of individual differences in each response (such as age, etc.), or which is most sensitive to dysfunction in PNS activity.

### 1.5. Age

There have been many studies on the effects of age on baseline pupil size [20,27,28,29,30,31,32]. All these studies found that baseline (or resting) pupil diameter decreases with age. The studies of Winn et al. [27], Rickman et al. [32], and Tekin et al. [30] also show that this age-related reduction in BPD reduced, but was still present, with increasing ambient light level.

The effect of age on the parameters of the PLR have been less well studied. A summary of germane results is given in Table 1. All the studies found that the rate of constriction (CV) was reduced with age. There is little consensus about the effects of age on the other parameters of the PLR. However, there appears to be some evidence that the latency to constriction (CL) may increase with age, and that measures of the amplitude of the PLR (CA and RCA) may decrease with age.

### 1.6. Anxiety and Trauma

Bakes, Bradshaw & Szabadi [33] examined the PLR in a sample of patients with a diagnosis of anxiety disorder and age-matched controls. No between-group differences were found for the baseline pupil diameter, but the patient group showed smaller pupil constrictions to the flash of light. This finding of reduced PLR with anxiety was extended to examine anxiety in a healthy population. Bitsios, Szabadi & Bradshaw [34] reported that the amplitude of the PLR was reduced as a function of both state and trait anxiety, both under “safe” conditions and “threat” conditions (where the person believed they may receive an electric shock). Nagai, Wada & Sunaga [35] found that high trait anxiety was related to greater BPD but smaller amplitude of constrictions. However, the study of Shioiri et al. [36] failed to find any effects of anxiety on these PLR parameters in both healthy controls and in remitted patients with panic disorder.

While there have been many studies on the effects of brain injury on the PLR [9], there have been few studies on the effect of post-traumatic stress disorder (PTSD) on the PLR. However, Mckinnon, Gray & Snowden [37] found that the amplitude of the PLR was reduced in patients with PTSD compared with controls.

### 1.7. Aims

The study had two main aims. First, to examine the reliability of two portable instruments ((a NeuroLight Algiscan R (ID-MED, Marseille, France) and an iPhone) using the Reflex-PLR Analyzer application (Brightlamp, Inc., Indianapolis, IN, USA) in a “field” setting, and to examine if they produced the same relationships between the PLR parameters as had been found under laboratory conditions where measurements were taken in dim light conditions and where the pupil was dark-adapted. To date, there appears to be no research on these important parameters under more “normal” light conditions or in field settings. The second aim was to assess the validity of the instruments by examining whether they could reproduce some well-established findings related to the PLR (such as the effect of age) and some other less-well-established findings (the effects of anxiety and PTSD symptomology). Measures of the PLR were taken in a relatively large (*N* > 100) sample of people that were tested outside of the laboratory where there would be a large variation in light levels, noise, distraction, etc., that is found in field settings.

## 2. Methods

Ethical permission for this study was awarded by the Ethics Committee of the Department of Psychology, Swansea University (Ref: 2021-5204-4285).

### Participants

Participants (*N* = 108) were recruited via a variety of methods in order to get variations in age, gender, ethnicity, etc. Adverts were placed on social media, inviting members of the general public to be tested and to contact the researchers. When contacted, the researchers then went to the location of the participants and either tested them in their own homes (*n* = 12) or in the back of the researcher’s campervan (*n* = 32). Some other participants were tested in the office of one of the researchers (*n* = 8) or at their desks/workplaces (*n* = 4). Finally, we also set up a table within the hallway of a busy Students’ Union building and invited passers-by to take part in the study (*n* = 52). Whilst conditions in terms of noise and the presence of others varied a great deal, we did limit the settings to areas that were light but were indoors to avoid direct sunlight.

The mean age of the sample was 32.1 years (SD = 14.4, range 18–68), with 43 males and 65 females. Eighty-four participants (77.8%) described themselves as “white”, 15 (13.9%) as “Asian”, 7 (6.5%) as “Black”, and 2 (1.8%) as “Other”. Eye colour was recorded in 70 of the participants. Of these, 42 (60.0%) described their eyes as “brown”, 22 (31.4%) as “blue”, and 6 (8.6%) as “green”. We did not take other measures, such as ophthalmic history, medication, or other diagnoses that might also affect PNS function. Such variations are part of the natural variation found in field settings which we wanted to maintain in this study. Hence, no participants were excluded from the study on these grounds.

## 3. Materials

### 3.1. NeuroLight

A NeuroLight (ID-MED Version 3.1, Marseille, France) portable infrared photometer was used to measure the PLR. The NeuroLight emits a flash of light and records the pupil size via an infrared camera at a rate of 60 Hz over a period of 3 s. Internal software then smooths the data and estimates the BPD in the period of 300 ms before the test flash. The software also estimates CL, CA, RCA, and CV. The readings were taken directly from the display after each measurement.

### 3.2. iPhone

PLRs were also taken using an iPhone 13 with the Reflex application (Brightlamp, Inc.). The iPhone produced a flash of light from the light-emitting diode on the back of the device, and the camera videoed the eye for a period of 5 s at a rate of 30 Hz. The Reflex software (Version 3.12.4) calculated the BPD in the period before the flash, the latency to start of constriction, the CA, and the maximum velocity of the constriction (CV_max_). These parameters were taken directly from the screen of the iPhone.

### 3.3. Anxiety

The State-Trait Anxiety Inventory (STAI) [38] consists of 20 items measuring current anxiety (state anxiety—10 questions) and usual baseline levels of anxiety for the individual (trait anxiety—10 questions). Each question is responded to on a four-point scale. In the present sample, both the state and trait scales showed good levels of reliability (Cronbach *α* = 0.88 and 0.85, respectively).

### 3.4. Trauma Symptomology

The International Trauma Questionnaire (ITQ) [39] consists of 18 questions that are scored on a five-point scale. From these scores, various indices can be extracted. For the purposes of this research, we were interested in the dimensional scales related to PTSD indicators. These were “re-experiencing in the here and now”, “avoidance score”, and the “sense of current threat”. In the present sample, the reliabilities of these scales were re-experiencing = 0.73, avoidance = 0.88, and threat = 0.67.

### 3.5. Procedure

Participants read an information sheet and were given a chance to ask questions about the study before giving written informed consent. They filled out a demographics form. For each of the instruments, we attempted to take three PLR measurements, with the order of testing (NeuroLight vs. iPhone) being counter-balanced across participants. Participants were asked to cover their non-tested eye with their right hand at the time of each measurement, and all measurements were taken in the left eye. They then completed the questionnaire measures, STAI, and ITQ, in that order.

### 3.6. Data Analysis and Statistical Analysis

To examine the reliability of each index of PLR, Chronbach’s alpha was calculated across the three readings for each instrument. For the descriptive and inferential statistics the three measurements were averaged. The distribution of scores for each PLR component and instrument met the assumptions of normality of distribution (all skews <1, all Kolmogorov–Smirnov tests *p* > 0.01) with the exception of the CV_max_ for the IP. For this variable, the data were transformed by taking the natural log for inferential statistics. Zero-order correlations between variables were assessed via Pearson’s correlation. To examine the effects of anxiety and PTSD, hierarchical multiple regressions were performed. At stage 1, age was entered. At stage 2, the scales of anxiety, or PTSD, were entered (after being z-scored [40]). Changes in the model fit and the standardised betas were assessed for statistical significance.

## 4. Results

For the majority of PLR parameters we were able to get three readings for all participants. However, when using the iPhone we were unable to get any parameter readings for one participant, unable to get CL readings for 11 participants, unable to get CV_max_ readings for 5 participants, and unable to get both CL and CV_max_ for one participant.

### 4.1. Reliability of Measures

The descriptive statistics for the PLR parameters are shown in Table 2. Most of the parameters achieved good levels of internal reliability (>0.70). The exception was for the parameter of constriction latency (CL) which was poor for the NeuroLight and extremely poor for the iPhone.

### 4.2. Correlations between the Instruments and Parameters

We next examined the relationships between the PLR parameters for each of the instruments in turn (Table 3). The results appeared similar across the two instruments. BPD was strongly related to CA, which was in line with many previous studies [7], while the measures of RCA and CV showed significant but much smaller relationships to the BPD and quite strong relationships to one another. The measure of CL was not related to any other parameter for either instrument. The correlations of each parameter between the two instruments are shown by the numbers on the diagonal (in bold). The parameters of BPD, CA, and CV were all significantly correlated across the instruments, whereas RCA and CL were not significant.

### 4.3. Effect of Age

The effect of age was examined via its first-order correlations with the PLR parameters. The results are shown in Table 4. Age was negatively correlated with BPD, CA, RCA, and CV. Age was not related to CL. On the whole, the correlations of the PLR parameters were greater for the NeuroLight instrument. A comparison of correlation coefficients showed significantly greater correlations with age for the NeuroLight measures compared with the iPhone measures for BPD (*Z*(106) = 4.61, *p* < 0.001) and the CA (*Z*(106) = 2.74, *p* = 0.003), but not for the other parameters.

### 4.4. Effect of Anxiety

To examine the effects of anxiety, the two scales of the STAI were regressed onto each of the PLR parameters in turn. As we suspected that STAI scores might be related to age (and zero-order correlations confirmed that both scales of the STAI were negatively related to age), we entered age into the regression equation at stage 1, and then the two scales of the STAI (z-scored [38]) at stage two.

The only PLR parameter that had significant change in the model fit when the STAI scales were introduced was the RCA measure of the NeuroLight (ΔR = 0.07, *p* = 0.019). Examination of the regression coefficients showed that trait anxiety was negatively predictive of RCA (*β* = −0.30, *p* = 0.005). Regression analysis without Step 1 (e.g., omitting age from the analysis) produced highly similar results. A similar pattern of results was found using the CA measure, but these did not achieve statistical significance.

### 4.5. Effect of PTSD Symptomology

A similar hierarchical regression was performed for the three (re-experiencing, avoidance, and threat) trauma scales of the ITQ. The addition of the three PTSD scales produced a modest increase in fit (*p* = 0.09). Examination of the regression coefficients showed that the Avoidance Scale was negatively predictive of RCA (*β* = −0.22, *p* = 0.03).

## 5. Discussion

The study examined if hand-held portable devices for the measurement of pupillometry could produce reliable and valid (in the sense that they could detect known relationships to other variables) results in a “field” setting. It also aimed to inform which of the possible parameters of the PLR might be most useful. Two implementations of pupillometry technology, a hand-held infra-red pupillometer (NeuroLight) and an iPhone using the Reflex application, were tested and compared.

There were some problems in getting reliable results from the iPhone Reflex application. First, some readings could not be taken for a small number of participants. Second, the measurement of CL showed very poor reliability. We speculate that this was due to a low sampling rate for this instrument and note that others have shown that a low sampling rate has caused similar problems in other systems designed to measure the PLR when the index involves precision in timing [29,41,42].

Most of the measurement parameters of the PLR showed good reliability. The relationship between the parameters was also consistent with previous studies. For example, most of the indexes of the PLR were strongly related to the baseline pupil size [5]. There were also strong correlations between CA, RCA, and CV, suggesting that these measurements are examining very similar processes of the PLR and could be used interchangeably.

### 5.1. Age

Increasing age was associated with smaller resting pupil sizes, a finding that has been well documented in laboratory studies under conditions of dark adaptation [20,28,29,43,44], and under the more normal light conditions [27,30,32,43] used here. Hence, the portable systems appear useful for the measurement of simple (static) pupil size.

We further found that the indices of PLR amplitude/movement (CA, RCA, and CV) were also reduced with age. Again, such findings have been previously reported under dark conditions [20,28,29,43,44] but have not been tested under “normal” lighting conditions. However, we found no support for the notion that latency changes with age during adulthood. Previous researchers have reported that constriction latency increases with age [43], while others did not find this effect [29], although no study appears to have examined these properties under light conditions. However, this “null” result should be taken with great caution due to the poor reliability of the CL measurement (particularly with the iPhone).

The findings of reduced PLR response with age therefore suggest that there is a reduction in PNS activity with age, which is in agreement with other sources of evidence [45].

### 5.2. Anxiety and PTSD Symptomology

Reduced PNS activity has been demonstrated in those with generalised anxiety disorders [46], in those with PTSD [16,47], and in those high on trait anxiety [48,49]. However, data on the relationship between these conditions and the PLR are relatively rare. Bakes et al. [33] showed that patients with generalised anxiety disorder showed reduced pupil amplitudes (CA) but no differences in baseline levels (BPD). This might imply that the RCA would also be different, but this was not calculated in the Bakes et al. study. Nagai et al. [35] also reported that constriction ratio (RCA in present terms) was also reduced as a function of trait anxiety in college students. The present results are in accord with these findings and extend them to conditions of natural lighting rather than in just the dark-adapted state.

McKinnon et al. [37] recently reported that the amplitude (CA) of the PLR was reduced in patients with a diagnosis of PTSD. In this study, the PLR was triggered by the presentation of a neutral image (one with no emotional content) whose luminance was matched to the blank (but bright) computer screen that preceded it. Hence, these conditions differ considerably from most standard PLR studies. The current findings, nevertheless, provide some support for the findings of McKinnon et al. The RCA (and, to some extent, the CA) was reduced in those people reporting high levels of PTSD related to “avoidance” symptoms. The present results differ somewhat in that McKinnon et al. found that all the symptoms (intrusions, avoidance, negative cognitions, and hyperarousal) were related to the reduction in CA, whereas the present study only found avoidant symptoms to be related. However, the studies differ in that the McKinnon et al. study tested patients with a formal diagnosis of PTSD (where presumably, symptoms of PTSD were relatively severe and persistent), whereas the present study looked at symptoms of PTSD in the general population, which may be sub-clinical in nature (as well as the differences in other procedures). Clearly, this is an area that needs further investigation given the potential of these measures, and particularly ones that can be taken quickly and cheaply in the “field”, to detect problems due to trauma and stress [50].

A limitation of the study was the large variation in conditions under which people were tested. For instance, changes in light level are known to alter the BPD and also the parameters of the PLR. Such random variations due to conditions will serve to add “noise” to the readings that might obscure more subtle influences arising due to individual differences such as levels of anxiety. However, these variations in conditions are similar to those that would be present if these instruments are to be used in the field. The sample was also one of convenience and it would be of interest to examine groups with clinical diagnoses, such as anxiety disorder or PTSD, using these instruments to calculate their ability to classify such disorders [5,6].

## 6. Conclusions

The present study showed that a hand-held portable infrared pupillometer can be used successfully to measure PLR and its parameters under field settings. However, we did not find the same level of support for the iPhone Reflex application (in line with other recent reports [21]), and suggest that further development of this technology is needed before it can be reliably used in these settings [22]. We hope that this attempt to show the practical usage of such an instrument can be used to guide others in using such technology to investigate a range of psychological and physical conditions related to parasympathetic nervous system dysfunction (for reviews see [5,7]) and other applied work [3,4,6,15].

## Figures and Tables

**Table 1:** Correlations between the pupillary light reflex parameters and age in previous studies.

	N	Age	BPD	CL	CA	RCA	CV
Straub et al. [26]	103 (48♀)	14–75	−0.61 *	0.42 *	-	-	−0.41 *
Piha & Halonen [29]	81 (44♀)	18–47	−0.44 *	-	-	−0.22	−0.52 *
Fotiou et al. [27]	100 (48♀)	18–81	−0.68 *	0.14	-	−0.39 *	−0.56 *^a^
Muddipi et al. [18]	79 (?♀)	20–84 ^b^	Negative * ^c^	-	Negative *	Positive *	Negative*
Tekin et al. [28]	155 (86♀)	6–64	−0.63 *	0.29 *	−0.08	-	−0.35 *

Note: BPD = baseline pupil diameter, CL = constriction latency, CA = constriction amplitude, RCA = relative constriction amplitude, CV = constriction velocity. a = Fotiou et al. reported this as a positive correlation but used a measure of where greater constriction speed was reported as a smaller negative number. b = estimated from graphs. c = no correlations are given other than to say their direction and significance. * *p* < 0.05.

**Table 2:** Descriptive statistics for the pupillary light reflex parameters.

	NeuroLight			iPhone		
	Mean	SD	Reliability	Mean	SD	Reliability
Baseline pupil diameter (mm)	5.64	1.13	0.97 **	5.53	1.09	0.93 **
Constriction latency (ms)	265.3	41.4	0.65 **	202.8	27.6	0.10
Constriction amplitude (mm)	1.95	0.59	0.92 **	1.41	0.66	0.85 **
Relative constriction amplitude (%)	34.0	6.6	0.90 **	24.5	8.2	0.78 **
Constriction velocity (mm/s) or maximum constriction velocity (mm/s^2^)	4.53	1.17	0.78 **	12.96	7.47	0.78 **

Note: ** *p* < 0.001.

**Table 3:** Correlations between the pupillary light reflex parameters. Numbers above the diagonal are for the NeuroLight measurements and those below the diagonal are for the iPhone measurements. The numbers on the diagonal (in bold) are for the correlation between the two instruments.

	1.	2.	3.	4.	5.
1. Baseline pupil diameter	0.66 **	0.03	0.81 **	0.33 **	0.48 **
2. Constriction latency	−0.09	**0.07**	−0.07	−0.15	−0.19
3. Constriction amplitude	0.76 **	−0.10	0.45 **	0.81 **	0.76 **
4. Relative constriction amplitude	0.47 **	−0.10	0.92 **	**0.21**	0.67 **
5. Constriction velocity or maximum constriction velocity	0.47 **	0.01	0.77 **	0.74 **	0.34 *

Note: * *p* < 0.01; ** *p* < 0.001.

**Table 4:** Correlations between the pupillary light reflex parameters and age.

	NeuroLight	iPhone
Baseline pupil diameter (mm)	−0.67 **	−0.37 **
Constriction latency (ms)	0.05	−0.15
Constriction amplitude (mm)	−0.57 **	−0.33 **
Relative constriction amplitude (%)	−0.27 *	−0.26 *
Constriction velocity or maximum constriction velocity	−0.47 **	−0.32 *

Note: * *p* < 0.01; ** *p* < 0.001.

## Data Availability

The datasets generated during and/or analyzed during the current study are available from the corresponding author on reasonable request.
